# Single-Molecule Magnet Properties in 3*d*4*f* Heterobimetallic Iron and Dysprosium Complexes Involving Hydrazone Ligand

**DOI:** 10.3390/molecules28176359

**Published:** 2023-08-30

**Authors:** Bertrand Lefeuvre, Thierry Guizouarn, Vincent Dorcet, Marie Cordier, Fabrice Pointillart

**Affiliations:** CNRS, ISCR (Institut des Sciences Chimiques de Rennes)—UMR 6226, University of Rennes, 35000 Rennes, France; bertrand.lefeuvre@univ-rennes1.fr (B.L.); thierry.guizouarn@univ-rennes1.fr (T.G.); vincent.dorcet@univ-rennes1.fr (V.D.); marie.dallon@univ-rennes1.fr (M.C.)

**Keywords:** dysprosium, iron, hydrazone, hydrazine, single-molecule magnet

## Abstract

The reaction between the ((*E*)-*N*′-(2-hydroxy-3-methoxybenzylidene)pyrazine-2-carbohydrazide) (H_2_opch) ligand and the metallo-precursor [Dy(hfac)_3_]·2H_2_O led to the formation of an homometallic coordination complex with the formula [Dy_2_(hfac)_3_(H_2_O)(Hopch)_2_][Dy(hfac)_4_] (**1**). In presence of both [Dy(hfac)_3_] 2H_2_O and the Fe(II) salt, the heterobimetallic tetranuclear [FeDy_3_(hfac)_8_(H_2_O)_2_(opch)_2_] (**2**) was isolated, while the addition of the co-ligand 1,2-Bis(2-hydroxy-3-methoxybenzylidene) hydrazine (H_2_bmh) led to the formation of two heterobimetallic tetranuclear complexes with the formula [Fe_3_Dy(hfac)_6_(opch)_2_(H_2_bmh)] C_6_H_14_ (**3**) **C_6_H_14_** and [Fe_2_Dy_2_(hfac)_7_(opch)_2_(H_2_bmh)] 0.5C_7_H_16_ (**4**) **0.5C_7_H_16_**. Single crystal X-ray diffraction and dc magnetic investigation demonstrated that **3** and **4** involved the iron center in the +II and +III oxidation states. Dynamic magnetic measurements highlighted the single-molecule magnet behavior of **1** and **2** in a zero applied dc field primarily due to the ferromagnetic interactions taking place in these compounds.

## 1. Introduction

Since the discovery of a Mn12 cluster [1] which displayed the first single-molecule magnet (SMM) behavior thirty years ago, the molecular magnetism field is still very active. Its activity was enhanced by the observation a decade later of similar slow magnetic relaxation for a mononuclear lanthanide complex [2]. After these two pioneer works, the community developed several chemical strategies to enhance the magnetic performances of the lanthanide-based SMMs to make them suitable candidates for applications in high-density data storage [3]. Thus, the lanthanide ions, such as Dy(III) and Tb(III), were associated with stable organic radicals to establish significant 2*p*4*f* magnetic exchange interactions, allowing the observation of blocking temperatures up to 30 K [4,5]. A few years later, theoretical work demonstrated that two coordinate dysprosium complexes with linear geometry are perfect to maximize the Ising magnetic anisotropy and the energy barrier value [6]. Such complexes with pseudo-linear coordination geometry were designed by chemists using organometallic chemistry [7,8,9], leading to an increase in the blocking temperature of up to 80 K [10]. Recently, the role of lanthanide—lanthanide bonds in the observation of huge coercive fields was reported [11]. The main drawback of these previously mentioned strategies is the poor stability in air and regarding the temperature. Consequently, chemists are also exploring the possibility of combining 3*d* and 4*f* elements in hetero-bimetallic complexes to exploit the strong magnetic anisotropy of the 4*f* ions and to establish significant 3*d*4*f* magnetic interactions, which can significantly lessen the quantum tunneling of magnetization (QTM) and improve the energy barrier [12]. The 3*d* ion can itself bring new physical properties, such as spin-crossover behavior. In this context, the combination of Fe(II) or Fe(III) with Ln(III) is of particular interest and it already permitted the design of 3*d*4*f* SMMs [13,14,15,16,17,18].

**Scheme 1:**
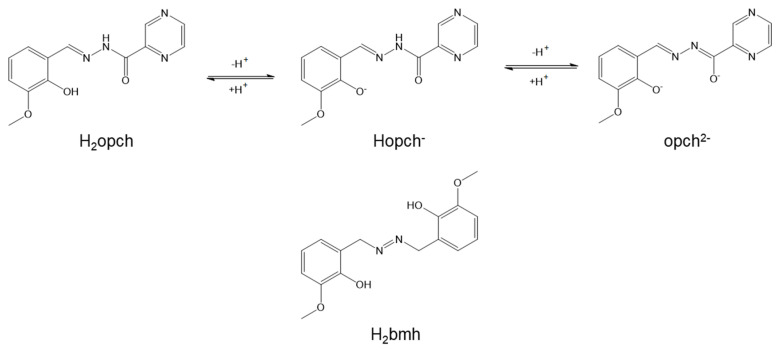
Molecular structures of the H_2_opch, Hopch^−^, opch^2−^ and H_2_bmh ligands.

The two ((*E*)-*N*′-(2-hybroxy-3-methoxybenzylidene)pyrazine-2-carbohydrazide) (H_2_opch) (Scheme 1) and 1,2-Bis(2-hydroxy-3-methoxybenzylidene) hydrazine (H_2_bmh) (Scheme 1) ligands were selected because they were used to design lanthanide SMMs [19,20,21,22] and Fe(III) spin-crossover complexes [23].

In this article, we proposed to engage these two ligands in coordination reactions with [Dy(hfac)_3_]·2H_2_O (hfac^−^ = 1,1,1,5,5,5-hexafluororacetylacetonate) and the Fe(II) salt. The homometallic trinuclear complex [Dy_2_(hfac)_3_(H_2_O)(Hopch)_2_][Dy(hfac)_4_] (**1**) as well as the three hetero-bimetallic tetranuclear complexes [FeDy_3_(hfac)_8_(H_2_O)_2_(opch)_2_] (**2**), [Fe_3_Dy(hfac)_6_(opch)_2_(H_2_bmh)]·C_6_H_14_ (**3**)·**C_6_H_14_** and [Fe_2_Dy_2_(hfac)_7_(opch)_2_(H_2_bmh)]·0.5C_7_H_16_ (**4**)·**0.5C_7_H_16_**, were isolated and characterized by single crystal X-ray diffraction. Both dc and ac magnetic properties were investigated and the role of the magnetic interaction was emphasized.

## 2. Results and Discussion

### 2.1. X-ray Structures

**Figure 1:**
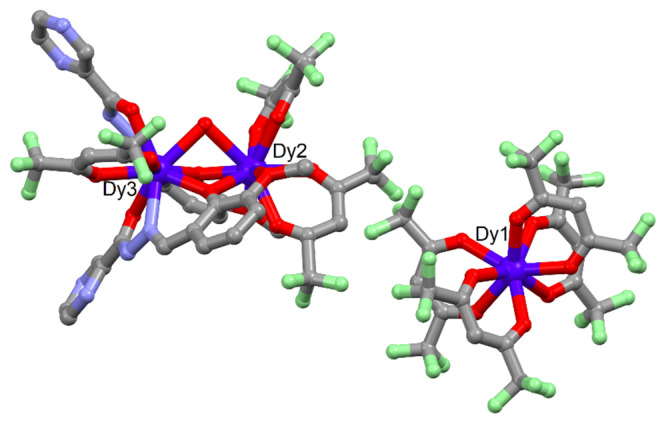
Experimental molecular structure of **1**. Hydrogen atoms are omitted for clarity. Code color: grey, C; blue, N; green, F; red, oxygen; dark blue, dysprosium.

**[Dy_2_(hfac)_3_(H_2_O)(Hopch)_2_]**·**[Dy(hfac)_4_]** (**1**). **1** crystallizes in the monoclinic space group P2_1_/n (N°14) (Figure 1, Supplementary Materials Figure S1, Table S1). The asymmetric unit is composed of one mono-cationic dinuclear complex, [Dy_2_(hfac)_3_(H_2_O)(Hopch)_2_]^+^, and one mono-anionic mononuclear complex, [Dy(hfac)_4_]^−^.

The Dy(III) center of the cation (Dy1) is coordinated with eight oxygen atoms coming from four hfac^-^ ancillary ligands. The Dy-O bond lengths are similar and range from 2.335(13) to 2.373(12) Å. The two Dy2 and Dy3 of the dimeric cation are coordinated to two deprotonated keto forms (Hopch^−^) (Scheme 1) of the ligand H_2_opch. Dy2 is linked to two Hopch^−^ ligands through the bischelating methoxyphenol moiety and its coordination sphere is filled by two hfac^−^ ancillary ligands and one bridging water molecule leading to a DyO9 surrounding. Furthermore, the Dy-O bond lengths range from 2.303(9) to 2.607(11) Å, and the Dy2-O distances involving a negatively charged oxygen atom are shorter (2.340 Å) than Dy2-O distances involving neutral oxygen atom (2.597 Å). The third Dy(III) (Dy3) is also coordinated to the two Hopch^−^ ligands through the trischelating coordination site [22] and its coordination sphere is filled with one hfac^-^ ancillary ligand and the bridging water molecule leading to a DyN2O7 surrounding. The Dy3-X bond lengths range from 2.308(9) to 2.540(11) Å. As expected, both Dy3-O involving neutral oxygen atoms and Dy3-N distances are longer than Dy3-O involving negatively charged oxygen atoms. The intra-molecular Dy2···Dy3 distance is 3.652 Å. The crystal packing highlighted the formation of a H-bond network (Figure S1) between the bridging H_2_O and the pyrazine (O37_w_···N89 = 2.681 Å) and the amido groups and the hfac^−^ anions of the [Dy(hfac)_4_]^−^ fragment (O7···N62 = 3.320 Å, O5···N62 = 3.339 Å and O1···N82 = 3.361 Å). The shortest inter-molecular Dy···Dy distance was found between Dy1 and Dy3 (7.990 Å).

**Figure 2:**
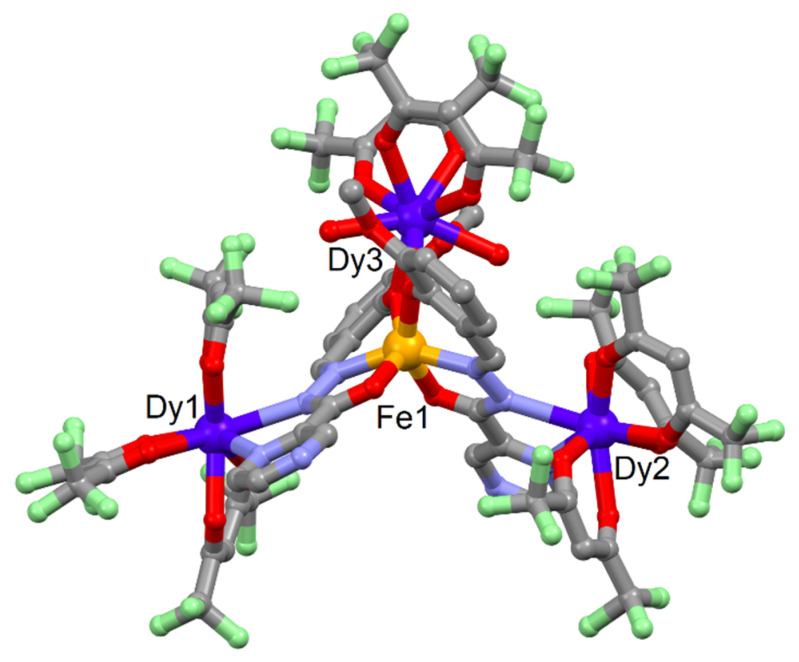
Experimental molecular structure of **2**. Hydrogen atoms are omitted for clarity. Code color: grey, C; blue, N; green, F; red, oxygen; orange, iron; dark blue, dysprosium.

**[FeDy_3_(hfac)_8_(H_2_O)_2_(opch)_2_]** (**2**). **2** crystallized in the monoclinic space group C2/c (N°15) (Figure 2 and Figure S2, Table S1). The asymmetric unit is composed of one heterobimetallic 3*d*4*f* tetranuclear complex with the formula [FeDy_3_(hfac)_8_(H_2_O)_2_(opch)_2_].

The Fe(III) ion is coordinated to two ligands in their enol forms (opch^2−^) (Scheme 1) through the tris-chelating site leading to a FeO4N2 surrounding with average Fe-O and Fe-N bond lengths of 1.999(7) Å and 2.118(9) Å, respectively. The two Dy(III) Dy1 and Dy2 adopted the same coordination sphere. They are coordinated to the bis-chelating site of opch^2−^ formed by the pyrazine and the hydrazone. The resulting DyO6N2 surrounding is obtained by the coordination of three hfac^-^ ancillary ligands. The third Dy(III) center is coordinated to the phenol moiety already coordinated to the Fe(III). To assume the neutrality of the tetranuclear complex, only two hfac^-^ ancillary ligands are coordinated to Dy3, while its coordination sphere is filled by two water molecules. Thus, the Dy3 adopted a DyO8 coordination sphere. The Dy-O distances are similar (2.351(8) Å) but shorter than the Dy-N distances (2.547(9) Å). Additionally, the shortest intramolecular Dy···Fe distance has been found between the two metal centers, which share the phenol oxygen atoms (3.542 Å), while the two other ones, Dy1···Fe (5.551 Å) and Dy2···Fe (5.576 Å), are longer because of the nature of the bridge. The crystal packing revealed the presence of hydrogen bond between the coordinated water and the pyrazine (O105_w_···N24 = 3.011 Å) (Figure S2). Moreover, the shortest inter-molecular Dy···Dy distance was found between the Dy2 (8.901 Å).

**Figure 3:**
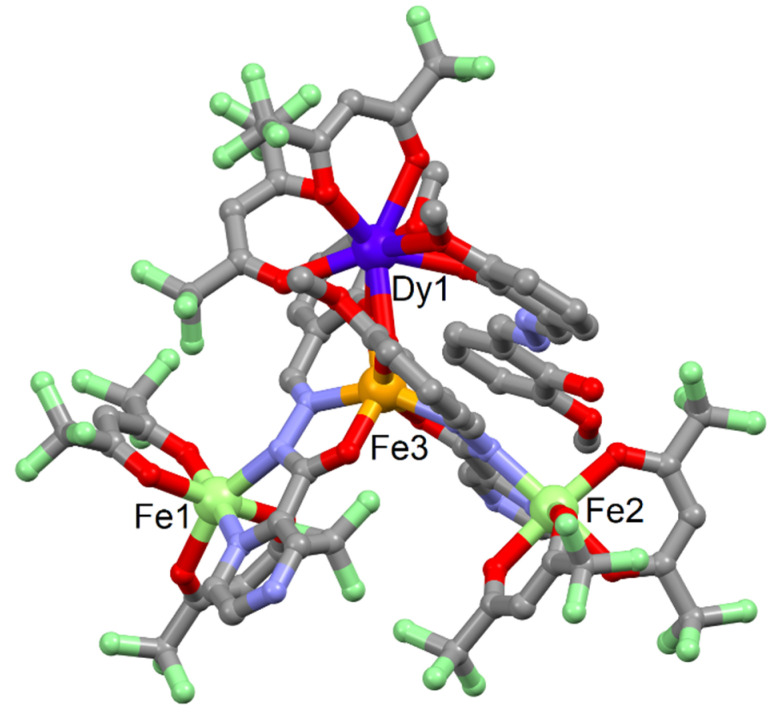
Experimental molecular structure of (**3**) C_6_H_14_. Hydrogen atoms and *n*-hexane molecules are omitted for clarity. Code color: grey, C; blue, N; green, F; red, oxygen; orange, trivalent iron; light green, divalent iron; dark blue, dysprosium.

**[Fe_3_Dy(hfac)_6_(opch)_2_(H_2_bmh)]**·**C_6_H_14_** (**3**)·**C_6_H_14_**. It crystallized in the triclinic space group P-1 (N°2) (Figure 3 and Figure S3, Table S1). The asymmetric unit is composed of one heterobimetallic 3*d*4*f* tetranuclear complex with the formula [Fe_3_Dy(hfac)_6_(opch)_2_(H_2_bmh)] and one disordered *n*-hexane molecule of crystallization.

As for compound **2**, the Fe(III) is coordinated to two opch^2−^ with a FeN2O4 surrounding. The two nitrogen bis-chelating coordination sites are occupied by two neutral [Fe(hfac)_2_] moieties, which are formed in situ during the coordination reaction. As expected, the Fe-O and Fe-N involving the Fe(II) ions (2.078(5) Å and 2.178(5) Å) are slightly longer than those involving the Fe(III) center (1.991(5) Å and 2.126(5) Å). Furthermore, a Dy(III) is coordinated to the two phenol groups and two hfac^-^ anions. In addition, instead of two water molecules (for **2**), a 1,2-Bis(2-hydroxy-3-methoxybenzylidene) hydrazine ligand (H_2_bmh) filled the coordination sphere of the Dy(III) ion, which adopts a DyO8 surrounding. Also, few intramolecular interactions take place, such as hydrogen bonds (O3···N2 = 2.709 Å and O1···O9 = 2.913 Å and O1···N1 = 2.552 Å) and π-π stacking between one of the opch^2−^ ligand and the H_2_bmh ligand. The shortest intramolecular Dy···Fe distance (Dy1···Fe3 = 3.584 Å) is shorter than the one for Fe···Fe (Fe2···Fe3 = 5.135 Å). The crystal packing also revealed intermolecular π-π stacking between the H_2_bmh ligands leading to the formation of a pseudo-dimer composed of tetranuclear complexes (Figure S3). These units interact through F···F and H···F contacts, and the shortest intermolecular Dy···Dy distance is 11.034 Å.

**Figure 4:**
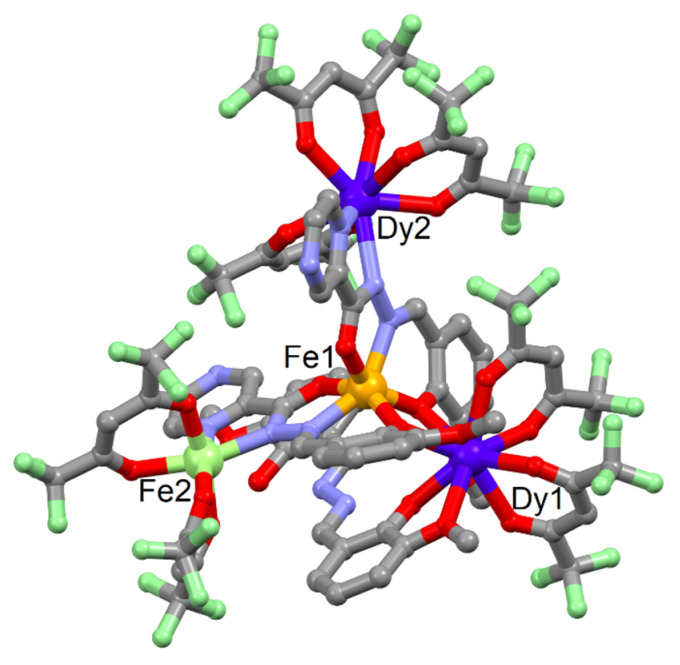
Experimental molecular structure of (**4**) 0.5C_7_H_16_. Hydrogen atoms and n-hexane molecules are omitted for clarity. Code color: grey, C; blue, N; green, F; red, oxygen; orange, trivalent iron; light green, divalent iron; dark blue, dysprosium.

**[Fe_2_Dy_2_(hfac)_7_(opch)_2_(H_2_bmh)]**·**0.5C_7_H_16_** (**4**)·**0.5C_7_H_16_**. It crystallized in the triclinic space group P-1 (N°2) (Figure 4 and Figure S4, Table S1). The asymmetric unit is composed of two heterobimetallic 3*d*4*f* tetranuclear complexes with the formula [Fe_2_Dy_2_(hfac)_7_(opch)_2_(H_2_bmh)] and one *n*-heptane molecule of crystallization. Complex **4** has some similitudes with **3**. Indeed, the main difference is the coordination of one [Dy(hfac)_3_] unit for **4** instead of one [Fe(hfac)_2_] for **3**. Based on the information from single crystal X-ray diffraction structures of **2** and **3**, i.e., the oxidation state +III of the iron center coordinate to the trischelating site of the opch^2−^ ligand, and to guarantee the electro-neutrality of the system, Fe1 and Fe2 centers have been associated to the +III and +II oxidation states, respectively. The crystal packing is not significantly affected by the same formation of pseudo-dimer of tetranuclear complexes (Figure S4). The shortest intermolecular Dy···Dy distance is also slightly shorter (9.495 Å) than for (**3**)·**C_6_H_14_.**

### 2.2. Magnetic Properties

#### 2.2.1. Static Magnetic Measurements

**Figure 5:**
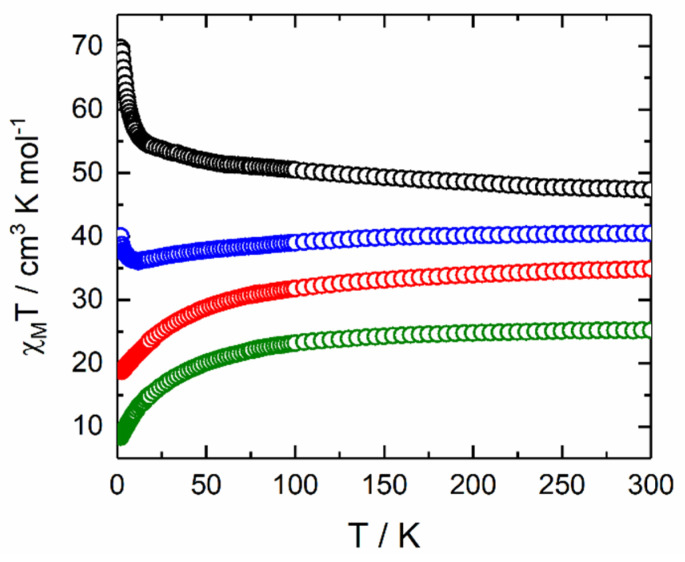
Temperature dependences of *χ_M_T* for **1** (blue), **2** (black), **3** (green) and **4** (red).

The temperature dependence of *χ_M_T* for samples **1**–**4** is represented in Figure 5. The room temperature values are 40.49 cm^3^·K·mol^−1^, 47.33 cm^3^·K·mol^−1^, 25.15 cm^3^·K·mol^−1^ and 34.87 cm^3^·K·mol^−1^ for **1**–**4**, respectively.

Such values are in agreement with expected values of 42.51 cm^3^·K·mol^−1^ (for three Dy(III) with a ^6^H_15/2_ ground state (GS) and g_J_ = 4/3), 46.89 cm^3^·K·mol^−1^ (for three Dy(III), ^6^H_15/2_ GS, g_J_ = 4/3 and one high spin state (HS) Fe(III)), 24.55 cm^3^·K·mol^−1^ (for one Dy(III), ^6^H_15/2_ GS, g_J_ = 4/3; one HS Fe(III) and two HS Fe(II)) and 35.72 cm^3^ K mol^−1^ (for two Dy(III), ^6^H_15/2_ GS, g_J_ = 4/3; one HS Fe(III) and one HS Fe(II)) [24,25]. In addition, upon cooling, *χ_M_T* decreases monotonically down to 8.36 cm^3^·K·mol^−1^ and 18.74 cm^3^ K mol^−1^ at 2 K for **3** and **4**, respectively. Such a decrease could be attributed to both thermal depopulation of the M_J_ states of the Dy(III) ion and antiferromagnetic interactions between the different spin carriers. For **1**, the *χ_M_T* slightly decreases until the temperature reaches 12 K due to the crystal field effect, while, for lower temperatures, the *χ_M_T* slightly increases the sign of the ferromagnetic interaction. Finally, **2** displays a continuous increase at low temperatures, reaching the value of 69.79 cm^3^·K·mol^−1^, which indicates the presence of significant ferromagnetic interactions. Magnetization for **1**–**4** is depicted in Figure S5 with experimental values of 14.84 Nβ at 50 kOe, 22.33 Nβ at 70 kOe, 8.42 Nβ at 70 kOe and 9.42 Nβ at 50 kOe, respectively.

#### 2.2.2. Dynamic Magnetic Measurements

The dynamic magnetic behavior was probed by measuring the in-phase (*χ_M_*′) and out-of-phase (*χ_M_*″) components of the ac susceptibility for compounds **1**–**4**. Such measurements were carried out using immobilized selected and crunched single crystals. Moreover, in the 1 Hz–10 kHz frequency range, no out-of-phase signal of the magnetic susceptibility was detected in the zero and the applied magnetic fields for both compounds **3** and **4**.

**Figure 6:**
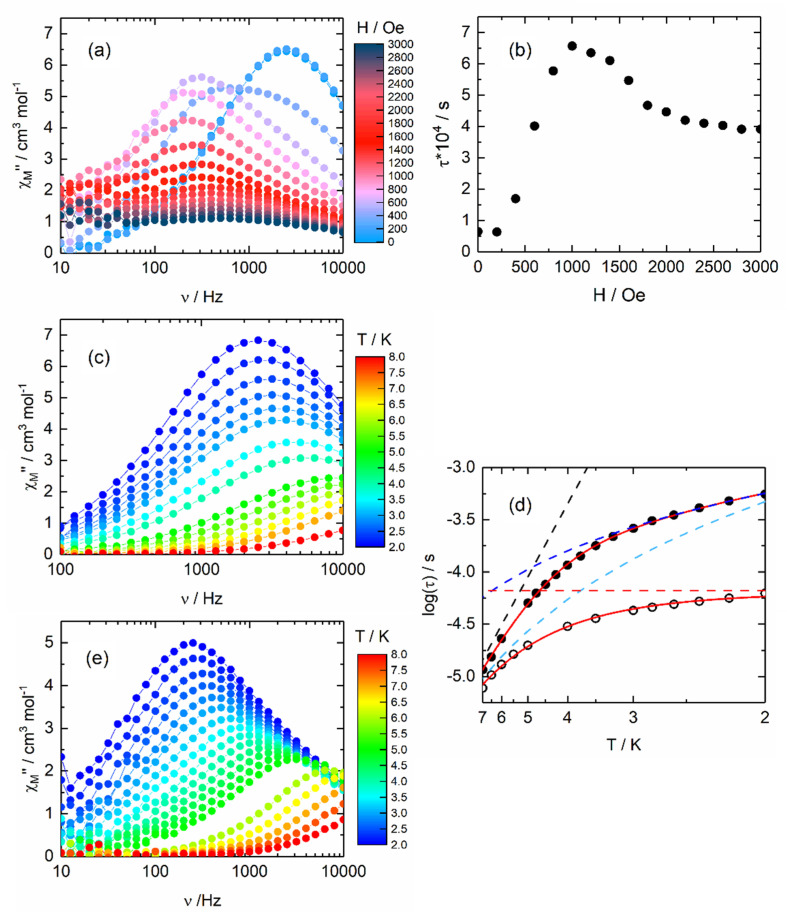
(**a**) Field dependence of the out-of-phase component of the magnetic susceptibility (*χ_M_*″) at 2 K in the field range of 0–3000 Oe for **1**. (**b**) Field dependence of the magnetic relaxation time at 2 K in the field range of 0–3000 Oe for **1**. (**c**) Out-of-phase component of the *ac* magnetic susceptibility data for **1** in zero fields in the temperature range of 2–8 K; (**d**) thermal dependence of the magnetic relaxation time for **1** in zero (open black circles) and 800 Oe (full black circles) applied magnetic fields in the 2–7 K temperature range. Full red lines are the best-fitted curves (see text), while the dashed lines are the Orbach (black), Raman (light and dark blue) and *QTM* (red) contributions. (**e**) Out-of-phase component of the *ac* magnetic susceptibility data for **1** in 800 Oe in the temperature range of 2–8 K.

**Figure 7:**
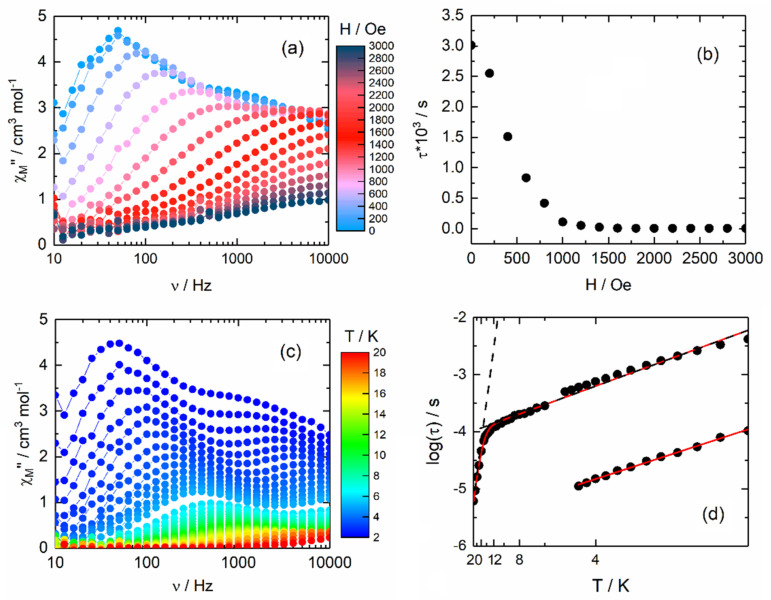
(**a**) Field dependence of the out-of-phase component of the magnetic susceptibility (*χ_M_*″) at 2 K in the field range of 0–3000 Oe for **2**. (**b**) Field dependence of the magnetic relaxation time at 2 K in the field range of 0–3000 Oe for **2**. (**c**) Out-of-phase component of the *ac* magnetic susceptibility data for **2** in zero field in the temperature range of 2–20 K. (**d**) Thermal dependence of the magnetic relaxation time for **2** in zero applied magnetic field in the 2–5 K and 2–20 K temperature ranges for HF and LF contributions, respectively. Full red lines are the best-fitted curves (see text), while the dashed black lines are the Orbach processes.

On the contrary, the two compounds **1** and **2** displayed a slow magnetic relaxation in the zero applied dc field (Figure 6a and Figure 7a). One of the possible explanations for the difference in magnetic behavior between **1** and **2** and **3** and **4** might be the lack of ferromagnetic interactions in **3** and **4**. A frequency dependence of the magnetic susceptibility in the temperature range of 2–8 K in the 100 Hz–10 kHz frequency window of the oscillating field was also observed at zero dc magnetic field for **1** (Figure 6c and Figure S6). An extended Debye model was used to extract the relaxation time (*τ*) [26,27,28] (Table S2), fitting simultaneously the two in-phase (*χ_M_*′) and out-of-phase (*χ_M_*″) components of the magnetic susceptibility. Furthermore, the Argand plot confirms that the observed slow magnetic relaxation corresponds to more than 90% of the sample (Figure S7). The corresponding thermal variation of the log(*τ*) is depicted in Figure 6d and could be fitted using the following equation (Equation (1)) for which the Orbach contribution was neglected:

(1) $$\tau^{-1}=\underset{Raman}{\underbrace{CT^{n}}}+\underset{Orbach}{\underbrace{\tau_{0}^{-1}\exp\left(-\frac{∆}{KT}\right)}}+\underset{QTM}{\underbrace{\tau_{TI}^{-1}}}$$

The best-fitted curves are represented in Figure 6d for C = 246.2(74) K^−*n*^ s^−1^ with *n* = 3.11(16) and *τ_TI_* = 6.62(35) × 10^−5^ s, where C and *n* are constant parameters for the Raman relaxation process and *τ_TI_* is the thermal independent relaxation time of the QTM. One could notice that the *n* constant parameter is lower than the expected *n* value for Kramers’ ions. Indeed, such a parameter value should be nine [29], but it is well known that, for molecular systems, lower values comprised between 2 and 7 could be found in the presence of both acoustic and optical phonons [30,31,32]. In order to enhance the magnetic performance of **1**, the *QTM* could be cancelled by applying an external dc field [33]. Thus, the field dependence of the magnetic susceptibility is studied (Figure 6a,b and Figure S8) and the relaxation time is extracted using the extended Debye model (Table S3). The application of a small magnetic field (<1000 Oe) led to the increase of the magnetic relaxation time (*τ*) (Figure 6b) due to the cancelling of the QTM, while, for a higher magnetic field value, *τ* decreases due to the direct process activation. To keep a significant *χ_M_*″ signal intensity, an 800 Oe value was selected. Under an applied field of H = 800 Oe, **1** highlighted a frequency dependence of the magnetization (Figure 6e and Figure S9). Both in-phase and out-of-phase signals of the magnetic susceptibility can be analyzed in the framework of the extended Debye model [26,27,28]. The temperature dependence of the relaxation time is plotted and depicted in Figure 6d (Table S4). At H = 800 Oe, it was determined by analyzing the normalized Argand (Figure S10) that more than 90% of the sample was involved in the slow magnetic relaxation. The thermal variation of the relaxation time could also be fitted considering a combination of Orbach and Raman processes. The best fit was obtained with *τ*_0_ = 1.50(16) × 10^−7^ s, Δ = 32.1(7) K and C = 496.3(26) K^−*n*^ s^−1^ with *n* = 1.83(6), where *τ*_0_ and Δ are the relaxation time and the energy barrier of the Orbach relaxation process (Figure 6d). One could remark that the 800 Oe external applied dc field cancelled efficiently the *QTM* and both Raman processes in 0 (light blue dashed line) and 800 Oe (dark blue dashed line) are similar as expected since such a process is field independent. It is also worth noticing that the Orbach process might be involved in the zero applied dc field but is almost negligible compared to the Raman and *QTM* processes, making it difficult to determine the relevant parameters for it.

Moving on, **2** displayed a non-zero *χ_M_*″ component sign of slow magnetic relaxation in a zero applied dc field at 2 K (Figure 7a). As soon as a dc field was applied, the maxima of the *χ_M_*″(ν) curve shifted to a higher frequency, as attested by the *τ* vs. H plot (Figure 7b). The *τ* values of the latter plot were extracted by fitting only the LF contribution of the magnetic susceptibility. The acceleration of the relaxation time of the magnetization could be due to the suppression of the ferromagnetic interaction under the applied dc field [12,34] and/or the appearance of a direct process. Since the best magnetic performances for **2** are observed under a zero applied dc field, the magnetic investigation was carried out only in such conditions. The thermal dependence of the magnetic susceptibility in a zero applied field showed two contributions with different intensities (Figure 7c and Figure S12). Such behavior might be attributed to the two Dy(III) centers with different surroundings i.e., one Dy(III) in a O8 environment, while the two others adopted a O6N2 environment. Thus, an extended Debye model considering two *τ* (see Supporting Information) was used (Table S6) for **2**. The temperature dependence of the relaxation time is plotted and depicted in Figure 7d for both low frequency (LF) and high frequency (HF) contributions.

The best fit of the thermal variation of the relaxation time for the HF contribution was obtained for an Orbach process only with *τ*^HF^_0_ = 2.01(11) × 10^−6^ s and Δ^HF^ = 8.0(2) K, while two Orbach processes were needed to fit the log(*τ*) vs. T plot for the LF contribution with *τ*^1^_0_ = 6.63(32) × 10^−5^ s and Δ^1^ = 9.0(2) K, *τ*^2^_0_ = 6.55(44) × 10^−10^ s and Δ^2^ = 184.4(12) K. The presence of two Orbach processes was already observed for a few SMMs involving Dy(III) in a N2O6 environment [35]. Moreover, such behavior was recently explained as a graphical problem for which the low temperature regime is close to a linear thermal dependence but, in fact, should correspond to a Raman process [36]. The absence of *QTM* in zero fields could also be attributed to the ferromagnetic interaction which has been identified in this complex.

## 3. Materials and Methods

### 3.1. Synthesis: General Procedures and Materials

The precursor Dy(hfac)_3_·2H_2_O (hfac^−^ = 1,1,1,5,5,5-hexafluoroacetonate anion), the 1,2-Bis(2-hydroxy-3-methoxybenzylidene) hydrazine (H_2_bmh) and ((*E*)-*N*′-(2-hyborxy-3-methoxybenzylidene)pyrazine-2-carbohydrazide) (H_2_opch) were synthesized following previously reported methods [19,20,37]. All other reagents were commercially available and used without further purification.

### 3.2. Synthesis of Complex [Dy_2_(hfac)_3_(H_2_O)(Hopch)_2_]·[Dy(hfac)_4_] (***1***)

To start, 82 mg of Dy(hfac)_3_·2H_2_O (0.1 mmol) and 27.1 mg of H_2_opch (0.1 mmol) were dissolved in 10 mL of CH_2_Cl_2_ and stirred at room temperature for 1 h. *n*-heptane was layered at room temperature in the dark. Slow diffusion leads to light yellow single crystals of (**1**), which are suitable for X-ray studies. The following measurements were reported: yield (determined from isolated single crystals) = 104.9 mg (42%). Anal. Calcd (%) for C_61_H_31_Dy_3_F_42_N_8_O_21_—C = 29.31, H = 1.24, N = 4.48; found—C = 29.09, H = 1.32, N = 4.41.

### 3.3. Synthesis of Complex [FeDy_3_(hfac)_8_(H_2_O)_2_(opch)_2_] (***2***)

For synthesis of complex **2**, 27.1 mg of H_2_opch (0.1 mmol) and 19.9 mg of FeCl_2_·4H_2_O (0.01 mmol) were dissolved in 20 mL of CH_3_CN. The solution turned dark brown after the addition of the iron salt. Then, a solution of 10 mL of CH_2_Cl_2_ containing 82 mg of Dy(hfac)_3_·2H_2_O (0.1 mmol) was added. The reaction was stirred for 1 h at room temperature and solvents evaporated under vacuum. Subsequently, 15 mL of CH_2_Cl_2_ was added to the residue. After filtration, diffusion of *n*-heptane to the filtrate led to the formation of dark brown single crystals suitable for X-ray studies. The following measurements were reported: yield (determined from isolated single crystals) = 85.9 mg (31%). Anal. Calcd (%) for C_66_H_28_Dy_3_F_48_N_8_O_24_—C = 28.57, H = 1.01, N = 4.04; found—C = 28.77, H = 1.09, N = 4.01.

### 3.4. Synthesis of Complex [Fe_3_Dy(hfac)_6_(opch)_2_(H_2_bmh)]·C_6_H_14_ (***3***)·C_6_H_14_

For synthesis of complex **3**, 27.1 mg of H_2_opch (0.1 mmol), 30 mg of H_2_bmh (0.1 mmol) and 82 mg of Dy(hfac)_3_·2H_2_O (0.1 mmol) were dissolved in 10 mL of CH_2_Cl_2_. Then, a solution of 10 mL of CH_3_OH containing 19.9 mg of FeCl_2_·4H_2_O (0.1 mmol) was added. The solution turned dark brown after the addition of the iron salt. The reaction was stirred for 1 h at room temperature and solvents evaporated under a vacuum. Following this, 15 mL of CH_2_Cl_2_ was added to the residue. After filtration, diffusion of *n*-hexane to the filtrate led to the formation of dark brown single crystals suitable for X-ray studies. The following measurements were reported: yield (determined from isolated single crystals) = 72.4 mg (29%). Anal. Calcd (%) for C_72_H_41_DyF_36_Fe_3_N_10_O_22_—C = 35.82, H = 1.70, N = 5.80; found—C = 35.89, H = 1.74, N = 5.88.

### 3.5. Synthesis of Complex [Fe_2_Dy_2_(hfac)_7_(opch)_2_(H_2_bmh)]·0.5C_7_H_16_ (***4***)·0.5C_7_H_16_

(**4**)·C_7_H_16_ was obtained from a protocol similar to (**3**)·C_6_H_14_, except that *n*-heptane was used instead of n-hexane for the crystallization process. The following measurements were reported: yield (determined from isolated single crystals) = 72.4 mg (21%). Anal. Calcd (%) for C_77_H_42_Dy_2_F_42_Fe_2_N_10_O_24_—C = 33.91, H = 1.54, N = 5.14; found—C = 33.80, H = 1.59, N = 5.18.

### 3.6. Crystallography

Single crystals of **1** and **2** were mounted on an APEXII Bruker-AXS diffractometer, while single crystals of **3** and **4** were mounted on an APEXIII D8 VENTURE Bruker-AXS diffractometer for data collection (MoK_α_ radiation source, λ = 0.71073 Å) received from the Centre de Diffractométrie (CDIFX), Université de Rennes, France (Table S1). A direct method using the SHELXT program [38] and a refined step with a full matrix least-squares method on F^2^ using the SHELXL-14/7 program [39] were used to solve and refine the structures. For structures containing large solvent accessible voids in which residual peaks of diffraction were observed, a SQUEEZE procedure of PLATON [40] was performed. Bond lengths, angles and atomic coordinates are included in CIF files for complete crystal structure results. These files are deposited as Supporting Information. CCDC numbers are 2281070 for **1**, 2281069 for **2**, 2281071 for (**3**)·C_6_H_14_ and 2281072 for (**4**)·0.5C_7_H_16_.

### 3.7. Physical Measurements

The Centre Régional de Mesures Physiques de l’Ouest (Rennes) performed the elemental analyses of the compounds. The dc magnetic susceptibility measurements were performed on solid polycrystalline samples with a Quantum Design MPMS-XL SQUID magnetometer between 2 and 300 K in applied magnetic fields of 200 Oe, 2000 Oe and 10,000 Oe for the 2–20, 20–80 K and 80–300 K temperature ranges, respectively. The microcrystallites are immobilized in a pellet made with Teflon tape. Quantum Design PPMS magnetometers were used to measure the ac magnetic susceptibility for frequencies between 10 and 10 kHz. Finally, these measurements were all corrected for the diamagnetic contribution, as calculated with Pascal’s constants.

## 4. Conclusions

The ligand ((*E*)-*N*′-(2-hyborxy-3-methoxybenzylidene)pyrazine-2-carbohydrazide) (H_2_opch) was used to design an homometallic Dy(III) dinuclear complex, [Dy_2_(hfac)_3_(H_2_O)(Hopch)_2_]·[Dy(hfac)_4_] (**1**), and three heterobimetallic 3*d*4*f* complexes: [FeDy_3_(hfac)_8_(H_2_O)_2_(opch)_2_] (**2**), [Fe_3_Dy(hfac)_6_(opch)_2_(H_2_bmh)] (**3**) and [Fe_2_Dy_2_(hfac)_7_(opch)_2_(H_2_bmh)] (**4**). The four compounds have been characterized by single crystal X-ray diffraction. The combination of dc magnetic investigations with the single-crystal X-ray diffraction structures allowed the identification of both Fe(II) and Fe(III) centers in the heterobimetallic 3*d*4*f* complexes **3** and **4**. The two compounds **1** and **2** displayed single-molecule magnet behavior in a zero applied dc field with magnetic relaxation occurring through thermally activated processes, such as Orbach and Raman. To end, our efforts to design heterobimetallic Fe(II/III)Dy(III) complexes with exciting magnetic properties, such as SMM and spin-crossover behavior, are in progress.

## Data Availability

Data are available from the authors.

## Supplementary Materials

The following supporting information can be downloaded at: https://www.mdpi.com/article/10.3390/molecules28176359/s1, Figure S1. Crystal packing of **1** highlighting the hydrogen bonds (dashed lines) between the cationic fragment [Dy_2_(hfac)_3_(H_2_O)(Hopch)_2_]^+^ and anionic [Dy(hfac)_4_]^−^ moieties. Figure S2. Crystal packing of **2** highlighting the hydrogen bonds between the pyrazine ring and coordinated water molecules of the neighboring complex. Figure S3. Crystal packing of **3** highlighting both intramolecular π-π stacking between the opch^2−^ and H_2_bmh ligands and intermolecular π-π stacking between the H_2_bmh ligands. Figure S4. Crystal packing of **4** highlighting both intramolecular π-π stacking between the opch^2−^ and H_2_bmh ligands and intermolecular π-π stacking between the H_2_bmh ligands. Figure S5. Field dependence of the magnetization at 2 K for **1** (blue), **2** (black), **3** (green) and **4** (red). Figure S6. Frequency dependence of the in-phase component of the magnetic susceptibility under zero applied magnetic field between 2 and 8 K for **1**. Figure S7. Normalized Cole–Cole plot for **1** at several temperatures between 2 and 7 K in zero applied magnetic field. Black lines are the best-fitted curves. Figure S8. In-phase component of the ac magnetic susceptibility for **1** at 2 K under a DC magnetic field from 0 to 3000 Oe. Figure S9. Frequency dependence of the in-phase component of the magnetic susceptibility under an applied magnetic field of 800 Oe between 2 and 8 K for **1**. Figure S10. Normalized Cole–Cole plot for **1** at several temperatures between 2 and 7 K under an applied magnetic field of 800 Oe. Black lines are the best-fitted curves. Figure S11. In-phase component of the ac magnetic susceptibility for 2 at 2 K under a DC magnetic field from 0 to 3000 Oe. Figure S12. Frequency dependence of the in-phase component of the magnetic susceptibility in zero applied magnetic fields between 2 and 20 K for **2**. Table S1: Summary of X-ray crystallographic data for **1**, **2**, (**3**)·C_6_H_14_ and (4)·0.5C_7_H_16_. Table S2: Best-fitted parameters (χ_T_, χ_S_, *τ* and α) with the extended Debye model for compound **1** at 0 Oe in the temperature range of 2–7 K. Table S3: Best-fitted parameters (χ_T_, χ_S_, *τ* and α) with the extended Debye model for compound **1** at 2 K in the magnetic field range of 0–3000 Oe. Table S4. Best-fitted parameters (χ_T_, χ_S_, *τ* and α) with the extended Debye model for compound **1** at 800 Oe in the temperature range of 2–7 K. Table S5. Best-fitted parameters (χ_T_, χ_S_, *τ* and α) with the extended Debye model for compound **1** at 2 K in the magnetic field range of 0–3000 Oe. Table S6. Best-fitted parameters (χ_T,1_, χ_S,1_, *τ*_1_, α_1_), (χ_T,2_, χ_S,2_, *τ*_2_ and α_2_) with the extended Debye model for compound **2** at 0 Oe in the temperature range of 2–20 K for LF contribution and 2–5 K for the HF.

## Sample Availability

Samples of the compounds are not available from the authors.
