# Supplementary material for: Single-Molecule Magnet Properties in 3d4f Heterobimetallic Iron and Dysprosium Complexes Involving Hydrazone Ligand
Source: Molecules. 2023 Aug 30;28(17):6359. doi: 10.3390/molecules28176359 (PMC10489976; doi:10.3390/molecules28176359)
Supplement: Supplementary file 1 [file molecules-28-06359-s001.zip › molecules-2542524-supplementary.pdf]

Supplementary Materials for

# Single-Molecule Magnet Properties in 3*d*4*f* Heterobimetallic Iron and Dysprosium Complexes Involving Hydrazone Ligand

**Bertrand Lefeuvre, Thierry Guizouarn, Vincent Dorcet, Marie Cordier and Fabrice Pointillart ***

CNRS, ISCR (Institut des Sciences Chimiques de Rennes)—UMR 6226, University of Rennes, 35000 Rennes, France

* Correspondence: fabrice.pointillart@univ-rennes1.fr; Tel.: +33-(0)223236752

**Table S1**. Summary of X-ray crystallographic data for **1**, **2**, (**3**)·$C_6H_{14}$ and (**4**)·0.5$C_7H_{16}$

| Compound | **1** | **2** |
|---|---|---|
| Empirical formula | $C_{61}H_{31}Dy_3F_{42}N_8O_{21}$ | $C_{66}H_{28}Dy_3F_{48}N_8O_{24}$ |
| Formula weight (g /mol) | 2497.44 | 2772.31 |
| CCDC number | 2281070 | 2281069 |
| Temperature (K) | 293(2) | 150(2) |
| Wavelength (Å) | 0.71073 | 0.71073 |
| Crystal size (mm) | 0.150 x 0.120 x 0.080 | 0.600 x 0.520 x 0.370 |
| Crystal system | monoclinic | monoclinic |
| Space group | *P2₁/n* | *C2/c* |
| *a* (Å) | 14.5929(19) | 41.643(2) |
| *b* (Å) | 29.356(4) | 15.6759(7) |
| *c* (Å) | 21.740(2) | 34.4062(16) |
| α (°) | 90 | 90 |
| β (°) | 103.093(3) | 123.308(2) |
| γ (°) | 90 | 90 |
| Volume (Å$^3$) | 9071.0(19) | 18770.5(16) |
| Z | 4 | 8 |
| $\rho$ (g.cm$^{-3}$) | 1.829 | 1.962 |
| Absorption coefficient (mm$^{-1}$) | 2.598 | 2.680 |
| *F (000)* | 6467 | 11888 |
| θ range for data collection (°) | 2.372 to 27.569 | 2.104 to 27.541 |
| Reflections unique | 65577 | 63848 |
| Reflections collected [I>2s(I)] | 20709 [R(int) = 0.1867] | 21424 [R(int) = 0.0822] |
| Data / restraints / parameters | 20709 / 899 / 1211 | 21424 / 152 / 1355 |
| Goodness-of-fit on *F*2 | 0.933 | 1.026 |
| Final R indices [I>2σ] | R1 = 0.0863, wR2 = 0.1867 | R1 = 0.0777, wR2 = 0.1685 |
| | | |

| Compound | (**3**)·$C_6H_{14}$ | (**4**)·0.5$C_7H_{16}$ |
|---|---|---|
| Empirical formula | $C_{78}H_{55}DyF_{36}Fe_3N_{10}O_{22}$ | $C_{161}H_{100}Dy_4F_{84}Fe_4N_{20}O_{48}$ |
| Formula weight (g /mol) | 2498.37 | 5552.00 |
| CCDC number | 2281071 | 2281072 |
| Temperature (K) | 150 | 150 |
| Wavelength (Å) | 0.71073 | 0.71073 |
| Crystal size (mm) | 0.250 x 0.150 x 0.080 | 0.560 x 0.250 x 0.150 |
| Crystal system | triclinic | triclinic |
| Space group | P-1 | P-1 |
| *a* (Å) | 15.495(3) | 17.430(3) |
| *b* (Å) | 15.860(3) | 24.201(4) |
| *c* (Å) | 21.900(4) | 25.909(4) |
| α (°) | 75.268(8) | 95.013(6) |
| β (°) | 84.448(7) | 102.018(7) |
| γ (°) | 65.881(6) | 103.401(6) |
| Volume ($Å^3$) | 4750.5(16) | 10292(3) |
| Z | 2 | 2 |
| $\rho$ ($g.cm^{-3}$) | 1.747 | 1.792 |
| Absorption coefficient ($mm^{-1}$) | 1.371 | 1.861 |
| *F (000)* | 16730 | 31106 |
| θ range for data collection (º) | 1.923 to 27.562 | 2.016 to 25.681 |
| Reflections unique | 65577 | 110359 |
| Reflections collected [I>2s(I)] | 21517 [R(int) = 0.0486] | 38832 [R(int) = 0.0554] |
| Data / restraints / parameters | 21517 / 414 / 1622 | 38832 / 1251 / 2771 |
| Goodness-of-fit on *F*2 | 1.140 | 1.120 |
| Final R indices [I>2σ] | R1 = 0.0635, wR2 = 0.1357 | R1 = 0.0846, wR2 = 0.2004 |

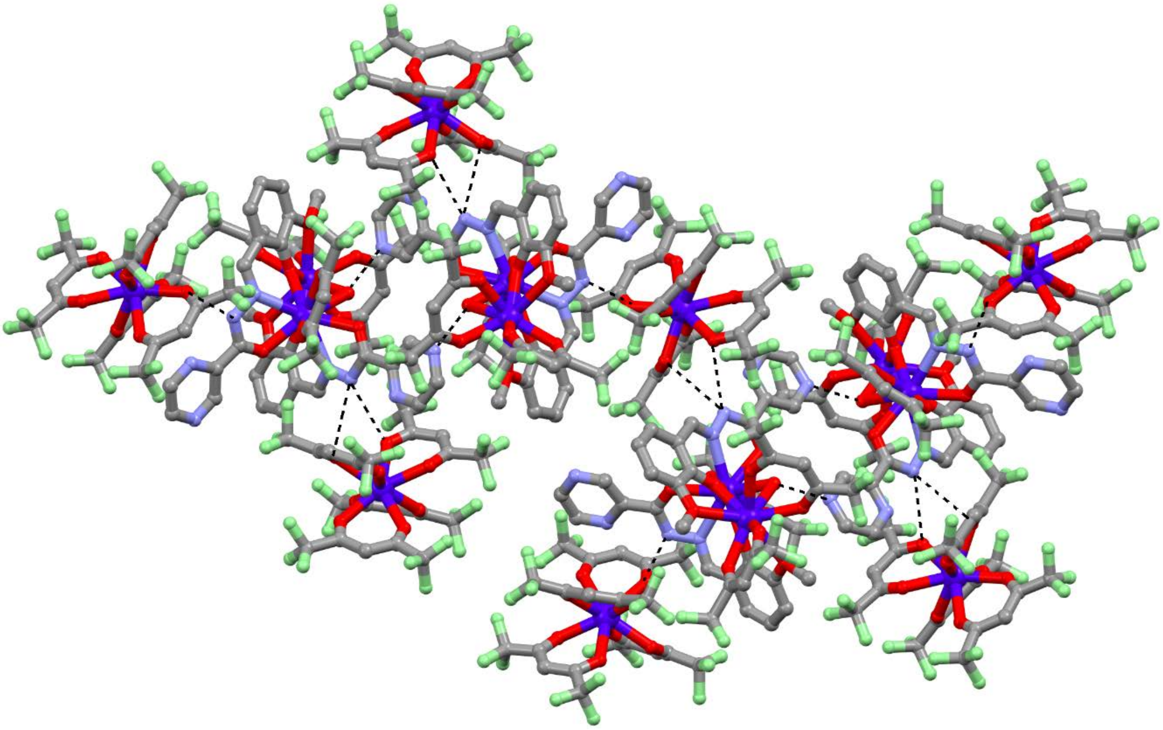

**Figure S1**. Crystal packing of **1** highlighting the hydrogen bonds (dashed lines) between the cationic fragment $[Dy_2(hfac)_3(H_2O)(Hopch)_2]^+$ and anionic $[Dy(hfac)_4]^-$ moieties.

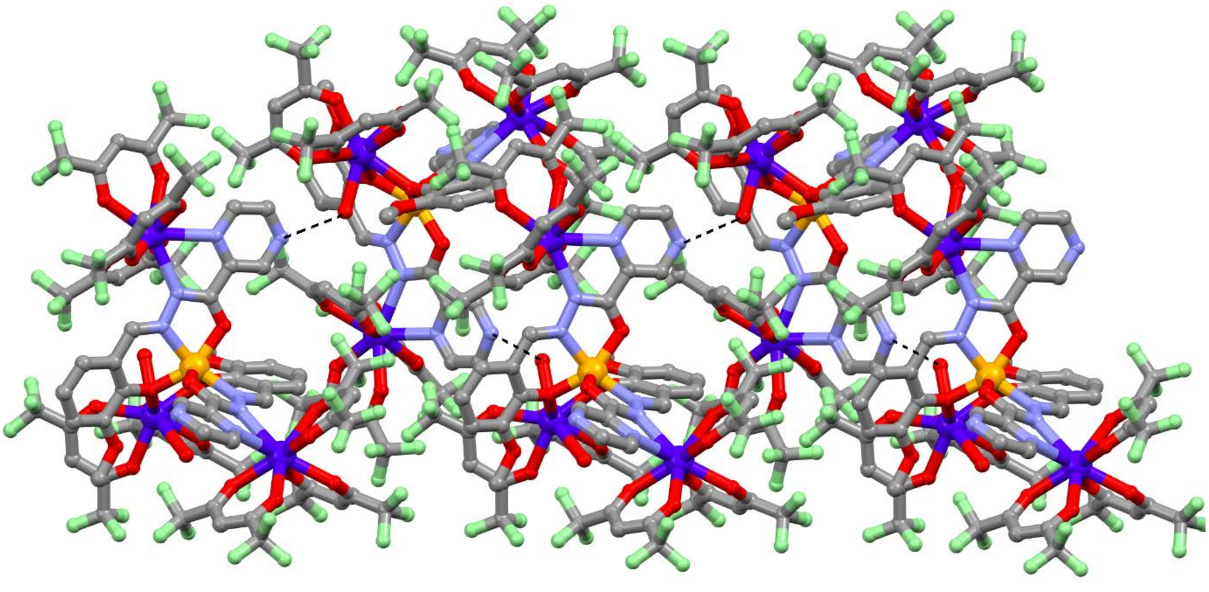

**Figure S2**. Crystal packing of **2** highlighting the hydrogen bonds between the pyrazine ring and coordinated water molecules of the neighboring complex.

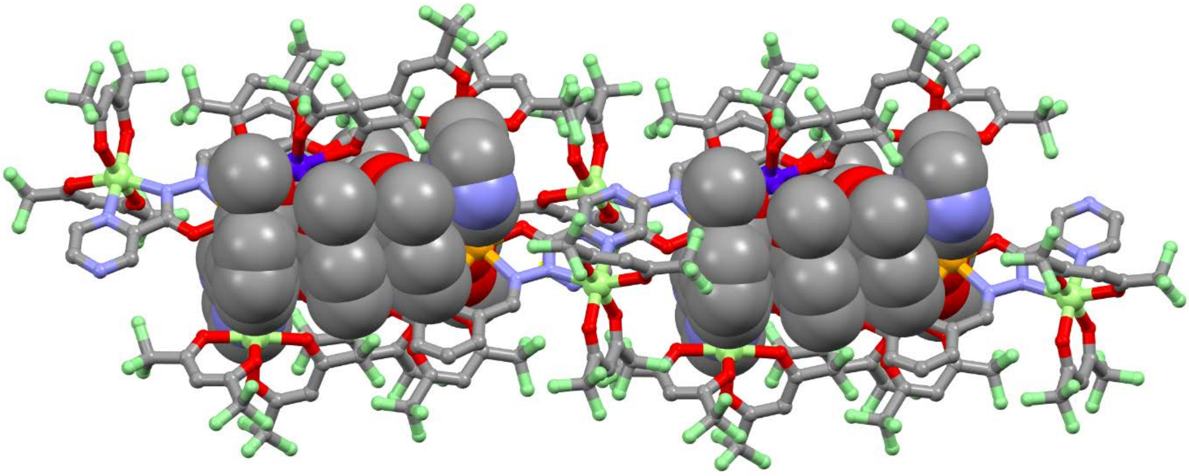

**Figure S3**. Crystal packing of **3** highlighting both intramolecular π-π stacking between the opch$^{2-}$ and $H_2$bmh ligands and intermolecular π-π stacking between the $H_2$bmh ligands.

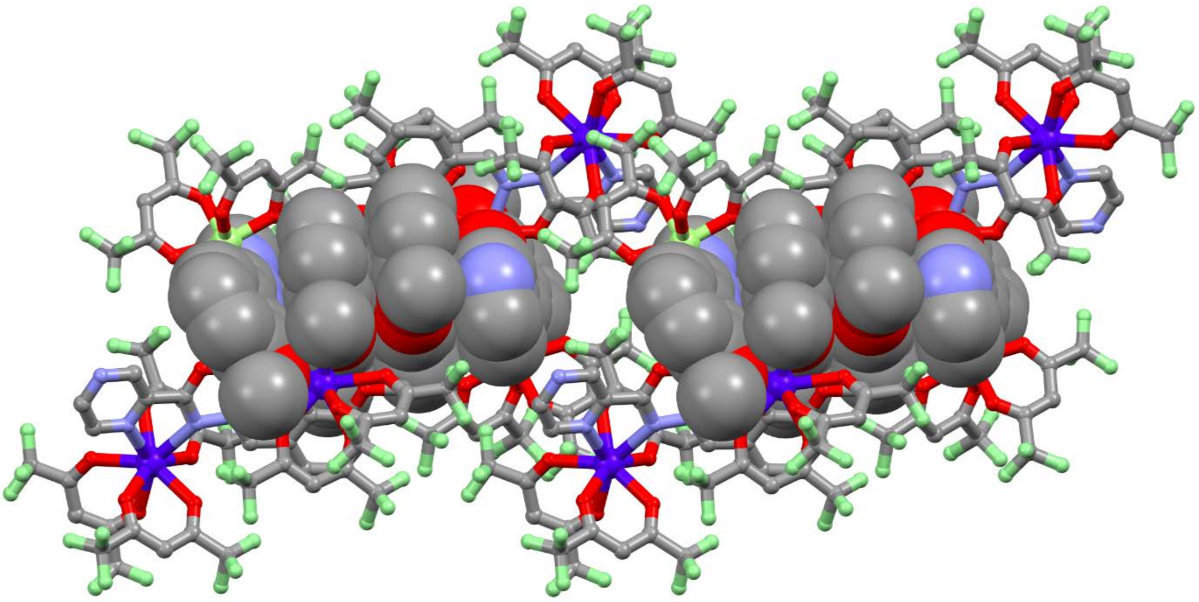

**Figure S4**. Crystal packing of **4** highlighting both intramolecular π-π stacking between the opch$^{2-}$ and $H_2$bmh ligands and intermolecular π-π stacking between the $H_2$bmh ligands.

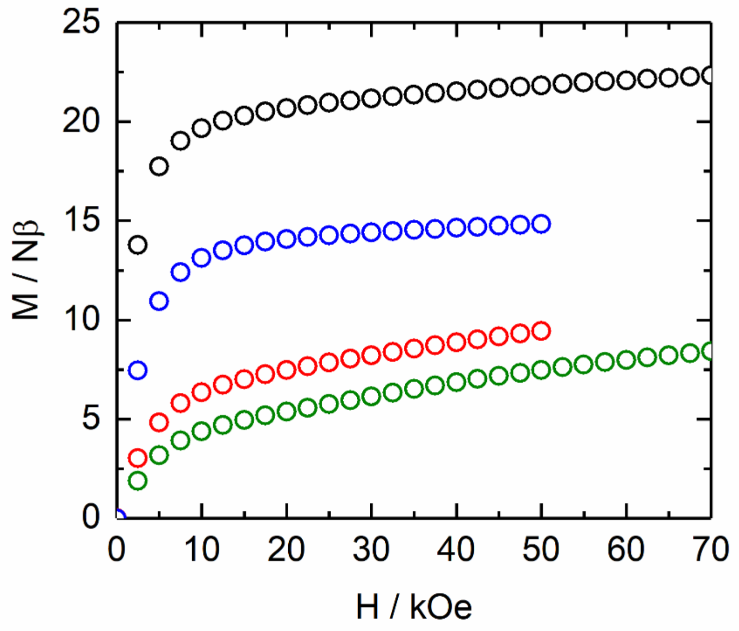


**Figure S5**. Field dependence of the magnetization at 2 K for **1** (blue), **2** (black), **3** (green) and **4** (red).

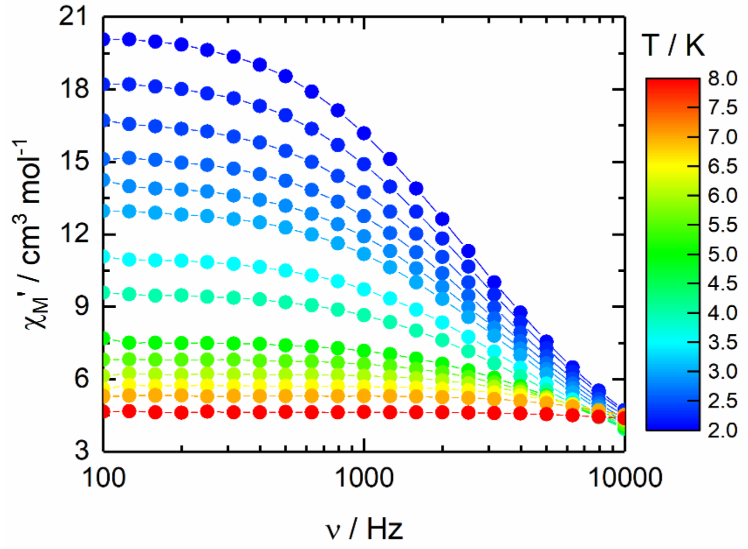


**Figure S6**. Frequency dependence of the in-phase component of the magnetic susceptibility under zero applied magnetic field between 2 and 8 K for **1**.

**Extended Debye model used for a single contribution.**

$$\chi_M' = \chi_S + (\chi_T - \chi_S)\frac{1+(\omega\tau)^{1-\alpha}\sin\left(\alpha\frac{\pi}{2}\right)}{1+2(\omega\tau)^{1-\alpha}\sin\left(\alpha\frac{\pi}{2}\right)+(\omega\tau)^{2-2\alpha}}$$

$$\chi_M'' = (\chi_T - \chi_S)\frac{(\omega\tau)^{1-\alpha}\cos\left(\alpha\frac{\pi}{2}\right)}{1+2(\omega\tau)^{1-\alpha}\sin\left(\alpha\frac{\pi}{2}\right)+(\omega\tau)^{2-2\alpha}}$$

With $\chi_T$ the isothermal susceptibility, $\chi_S$ the adiabatic susceptibility, $\tau$ the relaxation time and $\alpha$ an empiric parameter which describe the distribution of the relaxation time. For SMM with only one relaxing object $\alpha$ is close to zero. The extended Debye model was applied to fit simultaneously the experimental variations of $\chi_M$' and $\chi_M$'' with the frequency $\nu$ of the oscillating field ($\omega = 2\pi\nu$). Typically, only the temperatures for which a maximum on the $\chi$'' vs. *f* curves, have been considered. The best fitted parameters $\tau$, $\alpha$, $\chi_T$, $\chi_S$ are listed in Tables S2-S3 with the coefficient of determination $R^2$.

**Table S2.** Best fitted parameters ($\chi_T$, $\chi_S$, $\tau$ and $\alpha$) with the extended Debye model for compound **1** at 0 Oe in the temperature range 2-7 K.

| T / K | $\chi_S$ / cm$^3$ mol$^{-1}$ | $\chi_T$ / cm$^3$ mol$^{-1}$ | $\alpha$ | $\tau$ / s | $R^2$ |
|---|---|---|---|---|---|
| 2 | 1.92842 | 20.62981 | 0.18124 | 6.23787E-5 | 0.99961 |
| 2.2 | 1.49423 | 18.75713 | 0.19582 | 5.62229E-5 | 0.99964 |
| 2.4 | 1.40333 | 17.02381 | 0.19743 | 5.23913E-5 | 0.99951 |
| 2.6 | 1.35733 | 15.53114 | 0.1973 | 4.894E-5 | 0.99959 |
| 2.8 | 1.2692 | 14.36427 | 0.20284 | 4.58099E-5 | 0.9996 |
| 3 | 1.24015 | 13.25709 | 0.20093 | 4.26784E-5 | 0.99967 |
| 3.5 | 1.14889 | 11.21311 | 0.20467 | 3.5929E-5 | 0.99973 |
| 4 | 1.13843 | 9.68614 | 0.20073 | 3.00737E-5 | 0.9996 |
| 5 | 1.14093 | 7.62589 | 0.18134 | 1.98591E-5 | 0.99979 |
| 5.5 | 1.3178 | 6.85968 | 0.14196 | 1.62882E-5 | 0.99984 |
| 6 | 1.43913 | 6.2369 | 0.10188 | 1.30188E-5 | 0.99986 |
| 6.5 | 1.57958 | 5.73349 | 0.06554 | 1.03589E-5 | 0.99986 |
| 7 | 1.55121 | 5.31563 | 0.05244 | 7.72774E-6 | 0.9998 |

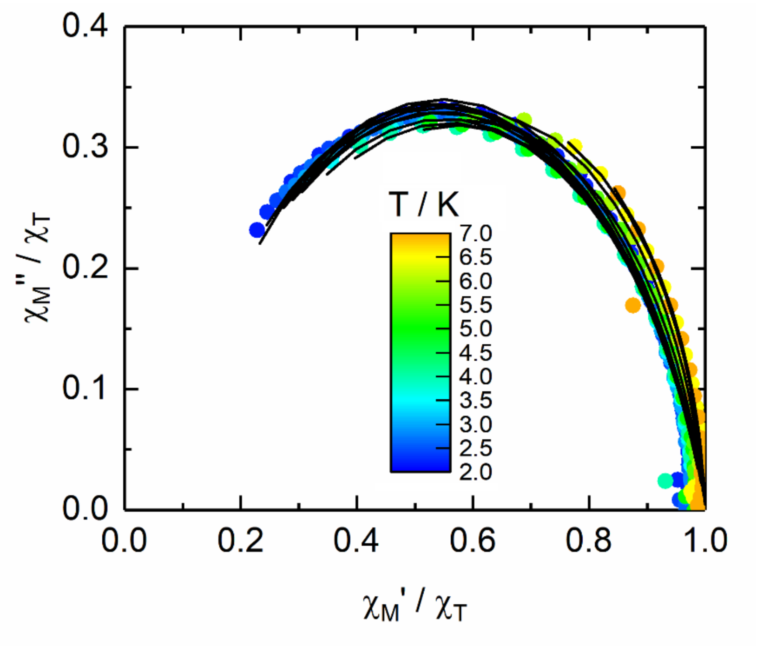


**Figure S7**. Normalized Cole-Cole plot for **1** at several temperatures between 2 and 7 K in zero applied magnetic field. Black lines are the best fitted curves.

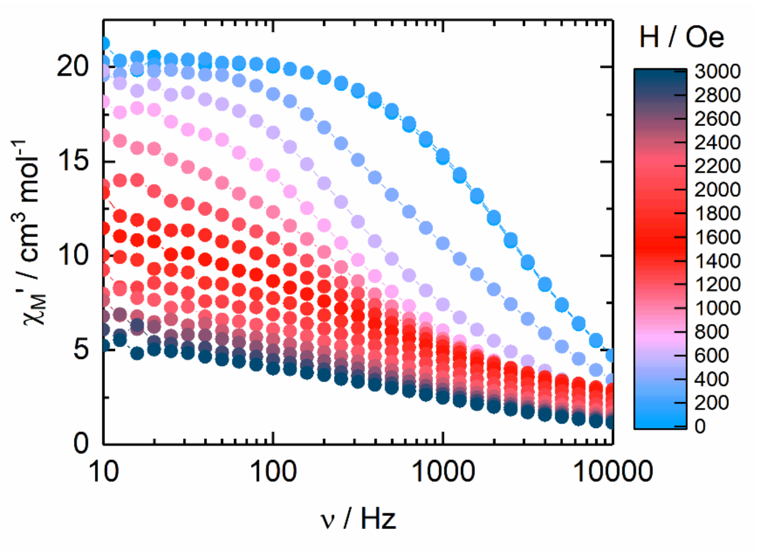


**Figure S8**. In-phase component of the ac magnetic susceptibility for **1** at 2 K under a DC magnetic field from 0 to 3000 Oe.

**Table S3.** Best fitted parameters ($\chi_T$, $\chi_S$, $\tau$ and $\alpha$) with the extended Debye model for compound **1** at 2 K in the magnetic field range 0-3000 Oe.

| H / Oe | $\chi_T$ / cm$^3$ mol$^{-1}$ | $\chi_S$ / cm$^3$ mol$^{-1}$ | $\alpha$ | $\tau$ / s | $R^2$ |
|---|---|---|---|---|---|
| 0 | 20.86186 | 0.95708 | 0.26259 | 6.49095E-5 | 0.99985 |
| 200 | 20.82455 | 1.05846 | 0.25225 | 6.3641E-5 | 0.99984 |
| 400 | 21.76533 | 0.40108 | 0.39782 | 1.69641E-4 | 0.99827 |
| 600 | 21.31089 | 0.95209 | 0.3697 | 4.01818E-4 | 0.99778 |
| 800 | 20.06306 | 0.89674 | 0.38672 | 5.77921E-4 | 0.99871 |
| 1000 | 18.00976 | 1.14523 | 0.42018 | 6.5705E-4 | 0.99927 |
| 1200 | 15.5595 | 1.67324 | 0.42653 | 6.35322E-4 | 0.9994 |
| 1400 | 14.06294 | 1.82066 | 0.45276 | 6.10698E-4 | 0.99968 |
| 1600 | 12.17587 | 1.66643 | 0.46158 | 5.47105E-4 | 0.99977 |
| 1800 | 10.83594 | 1.23646 | 0.48064 | 4.6796E-4 | 0.99978 |
| 2000 | 9.89583 | 0.78997 | 0.50342 | 4.46581E-4 | 0.99951 |
| 2200 | 8.8801 | 0.47652 | 0.51772 | 4.20152E-4 | 0.99908 |
| 2400 | 8.41515 | 0.18172 | 0.55171 | 4.1056E-4 | 0.99883 |
| 2600 | 7.60597 | 0.14751 | 0.54963 | 4.03767E-4 | 0.99948 |
| 2800 | 6.7484 | 0.03918 | 0.5599 | 3.91637E-4 | 0.99952 |
| 3000 | 6.25777 | 0 | 0.57665 | 3.91246E-4 | 0.99929 |

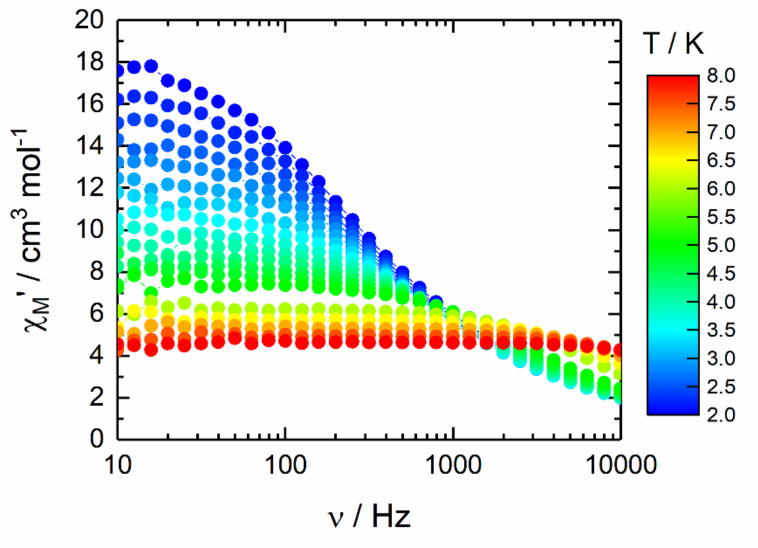


**Figure S9**. Frequency dependence of the in-phase component of the magnetic susceptibility under an applied magnetic field of 800 Oe between 2 and 8 K for **1**.

**Table S4.** Best fitted parameters ($\chi_T$, $\chi_S$, $\tau$ and $\alpha$) with the extended Debye model for compound **1** at 800 Oe in the temperature range 2-7 K.

| T / K | $\chi_S$ / cm$^3$ mol$^{-1}$ | $\chi_T$ / cm$^3$ mol$^{-1}$ | α | τ / s | $R^2$ |
|---|---|---|---|---|---|
| 2 | 1.09944 | 19.21907 | 0.36871 | 5.54789E-4 | 0.99917 |
| 2.2 | 1.11903 | 17.57512 | 0.35693 | 4.78093E-4 | 0.99923 |
| 2.4 | 1.12996 | 16.13961 | 0.34524 | 4.10416E-4 | 0.99901 |
| 2.6 | 1.1834 | 14.7959 | 0.32745 | 3.51162E-4 | 0.99887 |
| 2.8 | 1.11467 | 13.86769 | 0.32865 | 3.08662E-4 | 0.99913 |
| 3 | 1.10592 | 12.80409 | 0.31748 | 2.61005E-4 | 0.99894 |
| 3.25 | 1.02846 | 11.89991 | 0.31711 | 2.18022E-4 | 0.99918 |
| 3.5 | 0.99728 | 11.01663 | 0.30941 | 1.78653E-4 | 0.99885 |
| 3.75 | 0.93835 | 10.1657 | 0.30027 | 1.42153E-4 | 0.99945 |
| 4 | 0.99613 | 9.4461 | 0.27749 | 1.16302E-4 | 0.99871 |
| 4.25 | 1.05731 | 8.76824 | 0.25066 | 9.4013E-5 | 0.99761 |
| 4.5 | 0.84522 | 8.45381 | 0.27429 | 7.58919E-5 | 0.99839 |
| 4.75 | 0.94746 | 7.94927 | 0.24605 | 6.24077E-5 | 0.99726 |
| 5 | 0.992 | 7.48476 | 0.21956 | 5.03332E-5 | 0.99799 |
| 6 | 1.16855 | 6.24965 | 0.1467 | 2.29487E-5 | 0.99875 |
| 6.5 | 1.23564 | 5.7567 | 0.11572 | 1.53572E-5 | 0.99706 |
| 7 | 1.62468 | 5.32703 | 0.07139 | 1.16217E-5 | 0.99788 |

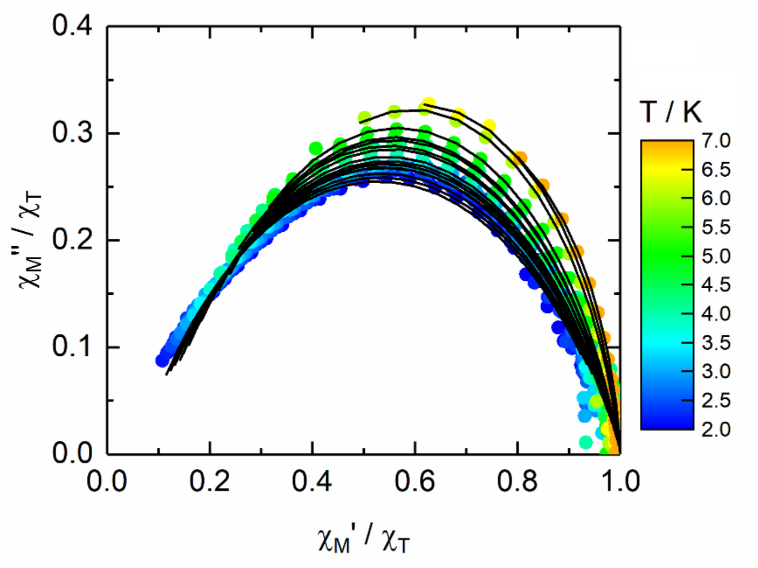


**Figure S10**. Normalized Cole-Cole plot for **1** at several temperatures between 2 and 7 K under an applied magnetic field of 800 Oe. Black lines are the best fitted curves.

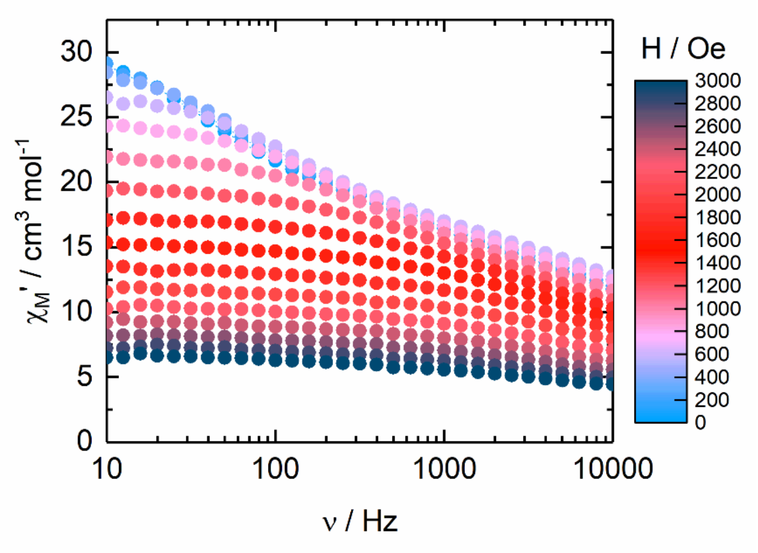


**Figure S11**. In-phase component of the ac magnetic susceptibility for **2** at 2 K under a DC magnetic field from 0 to 3000 Oe.

**Table S5.** Best fitted parameters ($\chi_T$, $\chi_S$, $\tau$ and $\alpha$) with the extended Debye model for compound **1** at 2 K in the magnetic field range 0-3000 Oe.

| H / Oe | $\chi_T$ / cm$^3$ mol$^{-1}$ | $\chi_S$ / cm$^3$ mol$^{-1}$ | α | τ / s | $R^2$ |
|---|---|---|---|---|---|
| 0 | 31.23619 | 16.55951 | 0.27248 | 1.2705E-4 | 0.99972 |
| 200 | 30.4294 | 16.82271 | 0.20708 | 9.92607E-5 | 0.99976 |
| 400 | 29.57228 | 15.51101 | 0.20296 | 6.58225E-5 | 0.99964 |
| 600 | 27.16268 | 14.73563 | 0.11452 | 3.10063E-5 | 0.99977 |
| 800 | 24.83925 | 12.94762 | 0.09647 | 1.86132E-5 | 0.99975 |
| 1000 | 22.71693 | 8.17951 | 0.12215 | 7.56254E-6 | 0.99951 |
| 1200 | 20.02087 | 6.30876 | 0.05381 | 2.84468E-6 | 0.99985 |
| 1400 | 17.59863 | 4.22725 | 0.05162 | 2.33048E-6 | 0.99982 |
| 1600 | 15.60979 | 1.14918 | 0.06609 | 2.1744E-6 | 0.9997 |
| 1800 | 13.68521 | 0 | 0.05691 | 1.74252E-6 | 0.99971 |
| 2000 | 11.96082 | 0.14259 | 0.05679 | 1.23955E-6 | 0.99988 |
| 2200 | 10.6229 | 0.23318 | 0.06231 | 1.50584E-6 | 0.99984 |
| 2400 | 9.47423 | 0 | 0.0545 | 1.33438E-6 | 0.99989 |
| 2600 | 8.4685 | 0 | 0.07513 | 1.95347E-6 | 0.9998 |
| 2800 | 7.63875 | 0 | 0.07538 | 2.06373E-6 | 0.99982 |
| 3000 | 6.9966 | 0 | 0.10321 | 2.94208E-6 | 0.99972 |

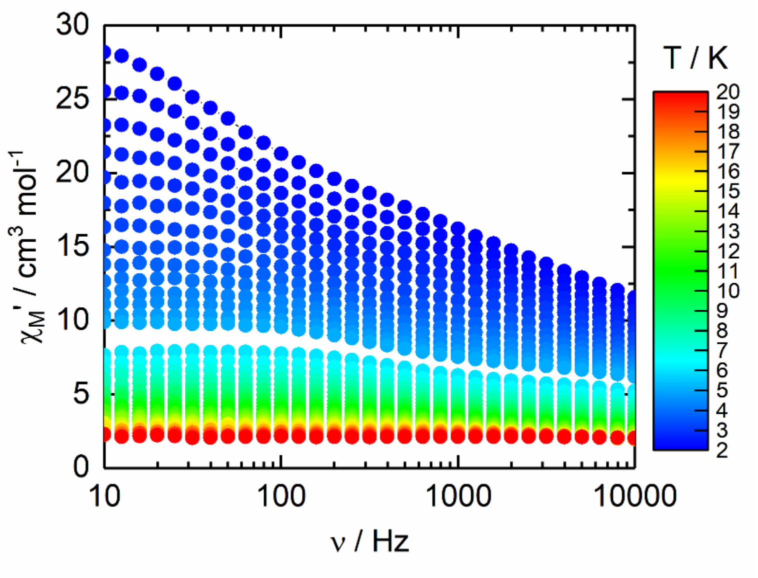


**Figure S12**. Frequency dependence of the in-phase component of the magnetic susceptibility in zero applied magnetic field between 2 and 20 K for **2**.

**Extended Debye model used for two relaxation contributions.**

$$\chi' = \chi_{S,tot} + (\chi_{T_1} - \chi_{S_1})\frac{1 + (\omega\tau_1)^{1-\alpha_1}\, sin\left(\frac{\pi\alpha_1}{2}\right)}{1 + 2(\omega\tau_1)^{1-\alpha_1}\, sin\left(\frac{\pi\alpha_1}{2}\right) + (\omega\tau_1)^{2-2\alpha_1}} +$$

$$+(\chi_{T_2} - \chi_{S_2})\frac{1+(\omega\tau_2)^{1-\alpha_2}\, sin\left(\frac{\pi\alpha_2}{2}\right)}{1+2(\omega\tau_2)^{1-\alpha_2}\, sin\left(\frac{\pi\alpha_2}{2}\right)+(\omega\tau_2)^{2-2\alpha_2}}$$

$$\chi'' = (\chi_{T_1} - \chi_{S_1})\frac{(\omega\tau_1)^{1-\alpha_1}\, cos\left(\frac{\pi\alpha_1}{2}\right)}{1 + 2(\omega\tau_1)^{1-\alpha_1}\, sin\left(\frac{\pi\alpha_1}{2}\right) + (\omega\tau_1)^{2-2\alpha_1}} +$$

$$+(\chi_{T_2} - \chi_{S_2})\frac{(\omega\tau_2)^{1-\alpha_2} cos\left(\frac{\pi\alpha_2}{2}\right)}{1+2(\omega\tau_2)^{1-\alpha_2}\, sin\left(\frac{\pi\alpha_2}{2}\right)+(\omega\tau_2)^{2-2\alpha_2}}$$

With $\chi_T$ the isothermal susceptibility, $\chi_S$ the adiabatic susceptibility, $\tau$ the relaxation time and $\alpha$ an empiric parameter which describe the distribution of the relaxation time. For SMM with only

one relaxing object $\alpha$ is close to zero. The extended Debye model was applied to fit simultaneously the experimental variations of $\chi_M$' and $\chi_M$'' with the frequency $\nu$ of the oscillating field ($\varpi$= 2$\pi\nu$). The best fitted parameters $\tau_1$, $\alpha_1$, $\chi_{1T}$, $\chi_{1S}$, $\tau_2$, $\alpha_2$, $\chi_{2T}$ and $\chi_{2S}$ are listed in Table S4 with the coefficient of determination $R^2$. $\tau_1$, $\alpha_1$, $\chi_{1T}$, $\chi_{1S}$ are parameters for the high frequency contribution while $\tau_2$, $\alpha_2$, $\chi_{2T}$ and $\chi_{2S}$ are for the low frequency contribution.

**Table S6.** Best fitted parameters ($\chi_{T,1}$, $\chi_{S,1}$, $\tau_1$, $\alpha_1$), ($\chi_{T,2}$, $\chi_{S,2}$, $\tau_2$ and $\alpha_2$) with the extended Debye model for compound **2** at 0 Oe in the temperature range of 2-20 K for LF contribution and 2-5 K for the HF.

| T / K | $\chi_{T,1}$ / cm$^3$ mol$^{-1}$ | $\chi_S$ / cm$^3$ mol$^{-1}$ | $\tau_1$ / s | $\alpha_1$ | $\chi_{T,2}$ / cm$^3$ mol$^{-1}$ | $\alpha_2$ | $\tau_2$ / s | $R^2$ |
|---|---|---|---|---|---|---|---|---|
| 2 | 23.14681 | 7.44071 | 1.03905E-4 | 0.5339 | 14.30059 | 0.08029 | 0.0042 | 0.99997 |
| 2.2 | 20.54606 | 7.50947 | 8.00474E-5 | 0.49913 | 13.56112 | 0.04855 | 0.00336 | 0.99995 |
| 2.4 | 17.81618 | 7.49697 | 5.44898E-5 | 0.44314 | 13.56253 | 0.07123 | 0.00262 | 0.99996 |
| 2.6 | 15.93495 | 7.38695 | 4.29753E-5 | 0.39511 | 13.08898 | 0.07606 | 0.00211 | 0.99995 |
| 2.8 | 14.77667 | 6.97594 | 3.65369E-5 | 0.38485 | 11.90137 | 0.04286 | 0.00175 | 0.99991 |
| 3 | 13.5505 | 6.69934 | 3.05859E-5 | 0.35794 | 11.24398 | 0.04206 | 0.00145 | 0.9999 |
| 3.25 | 12.21672 | 6.32771 | 2.424E-5 | 0.32977 | 10.60861 | 0.06133 | 0.00119 | 0.99992 |
| 3.5 | 11.28753 | 5.90759 | 2.07682E-5 | 0.32569 | 9.6693 | 0.04345 | 0.00101 | 0.99983 |
| 3.75 | 10.33059 | 5.51819 | 1.69304E-5 | 0.31115 | 9.05219 | 0.06274 | 8.52474E-4 | 0.99993 |
| 4 | 9.68373 | 5.1441 | 1.50088E-5 | 0.32144 | 8.29537 | 0.04719 | 7.53709E-4 | 0.99988 |
| 4.25 | 8.99547 | 4.80999 | 1.27977E-5 | 0.31675 | 7.72805 | 0.05449 | 6.59421E-4 | 0.9999 |
| 4.5 | 8.35998 | 4.56554 | 1.11256E-5 | 0.30586 | 7.42211 | 0.08543 | 5.99685E-4 | 0.9999 |
| 4.75 | 7.98333 | 4.3132 | 1.08919E-5 | 0.31816 | 6.74214 | 0.04119 | 5.39284E-4 | 0.99991 |
| 5 | 7.5394 | 4.18553 | 1.05966E-5 | 0.31506 | 6.49232 | 0.04491 | 5.01887E-4 | 0.99988 |
| 6 | | 5.44871 | | | 8.01354 | 0.16459 | 2.85236E-4 | 0.99988 |
| 6.5 | | 4.98552 | | | 7.31706 | 0.16846 | 2.61111E-4 | 0.99986 |
| 7 | | 4.51619 | | | 6.72845 | 0.19539 | 2.26393E-4 | 0.99989 |
| 7.5 | | 4.18869 | | | 6.20026 | 0.1882 | 2.08908E-4 | 0.99989 |
| 8 | | 3.95811 | | | 5.74466 | 0.15757 | 2.01724E-4 | 0.99987 |
| 8.5 | | 3.71855 | | | 5.35852 | 0.14844 | 1.92377E-4 | 0.99987 |
| 9 | | 3.44582 | | | 5.01504 | 0.16449 | 1.70101E-4 | 0.99986 |
| 9.5 | | 3.23919 | | | 4.71603 | 0.16576 | 1.59406E-4 | 0.99989 |
| 10 | | 3.05424 | | | 4.47706 | 0.1796 | 1.53085E-4 | 0.99988 |
| 10.5 | | 2.88668 | | | 4.22681 | 0.17322 | 1.39455E-4 | 0.99983 |
| 11 | | 2.75007 | | | 4.04119 | 0.1827 | 1.37622E-4 | 0.99973 |
| 11.5 | | 2.61799 | | | 3.80765 | 0.16475 | 1.24736E-4 | 0.99978 |
| 12 | | 2.51885 | | | 3.64103 | 0.15195 | 1.21892E-4 | 0.99984 |
| 12.5 | | 2.42113 | | | 3.49851 | 0.15255 | 1.19075E-4 | 0.99962 |
| 13 | | 2.27394 | | | 3.35305 | 0.18117 | 1.04032E-4 | 0.99988 |
| 13.5 | | 2.20268 | | | 3.18879 | 0.14399 | 9.59164E-5 | 0.99955 |
| 14 | | 2.11989 | | | 3.07783 | 0.14822 | 8.98896E-5 | 0.99971 |
| 14.5 | | 2.04662 | | | 2.96624 | 0.13927 | 8.20713E-5 | 0.99987 |
| 15 | | 1.926 | | | 2.89683 | 0.19175 | 6.954E-5 | 0.99974 |
| 16 | | 1.78063 | | | 2.67981 | 0.1573 | 4.57622E-5 | 0.99955 |
| 17 | | 1.63358 | | | 2.50439 | 0.1338 | 2.57731E-5 | 0.99979 |
| 18 | | 1.56345 | | | 2.34464 | 0.0518 | 1.61296E-5 | 0.99955 |

| | | | | | | | | |
|---|---|---|---|---|---|---|---|---|
| 19 | | 1.45639 | | | 2.22803 | 0.03678 | 9.30854E-6 | 0.99992 |
| 20 | | 1.41943 | | | 2.11159 | 0.01291 | 6.10194E-6 | 0.99994 |
